# Nutrition literacy among primary school students in Nanshan District, Shenzhen: current status and influencing factors

**DOI:** 10.3389/fnut.2026.1775403

**Published:** 2026-03-03

**Authors:** Jing Yang, Nana Li, Ti Zhang, Xiaoyue Li, Liuyuan Zheng, Minmin Zhu

**Affiliations:** 1 Shenzhen Nanshan Center for Chronic Disease Control, Shenzhen, China; 2 School of Public Health, Zunyi Medical University, Zunyi, China

**Keywords:** health literacy, influencing factors, nutrition literacy, primary school students, Shenzhen

## Abstract

**Objective:**

Nutrition literacy is critical for establishing healthy dietary behaviors during childhood, yet research on this topic among primary school students in rapidly urbanizing China remains limited. The aim of this large-scale survey is to assess the current status and identify key influencing factors of nutrition literacy among primary school students in Nanshan District, Shenzhen City.

**Methods:**

A cross-sectional survey was conducted in October 2024 utilizing a cluster random sampling method. A total of 2,423 students from 21 public primary schools participated. Data were collected using the validated “Nutrition Literacy and Dietary Behavior Questionnaire for School-aged Children”, which evaluated four dimensions: nutrition knowledge and concepts, food selection, food preparation, and food intake. Statistical analyses included descriptive statistics, correlation analysis, and binary logistic regression.

**Results:**

Participants achieved a mean total nutrition literacy score of 69.93 ± 8.75, with 30.38% meeting the criterion for adequate nutrition literacy (score ≥75). Interdimensional analysis revealed statistically significant positive correlations among all four domains (*r* = 0.198 ~ 0.363, *p* < 0.001). Multivariable logistic regression identified grade level—representing individual-level factors (Grade 5 vs. Grade 3 OR = 0.626, 95% CI: 0.500–0.783), high household economic status—family-level factors (OR = 1.389, 95% CI: 1.139–1.649), and participation in school activities including nutrition-related activities (OR = 1.346, 95% CI: 1.125–1.611) and regular weight monitoring (OR = 1.346, 95% CI: 1.125–1.611) as key predictors of adequate nutrition literacy.

**Conclusion:**

Nutrition literacy among primary school students in Nanshan District requires substantial improvement and is influenced by factors at individual, familial, and institutional levels. These findings suggest the necessity of developing a comprehensive, student-centered intervention model that integrates family-school collaboration to effectively enhance nutritional literacy.

## 1 Introduction

Guided by the Healthy China 2030 initiative, the nutritional health status of the population has become a key indicator for measuring national social development and public well-being (1). Childhood and adolescence represent a critical period in the life course, where nutritional status not only influences individual growth and cognitive development but also serves as a foundational element impacting a nation’s future human capital quality and public health burden (2). During this crucial stage, alongside traditional dietary intake, “nutrition literacy” is increasingly emerging as a central focus in health promotion. It extends far beyond the accumulation of static knowledge, emphasizing a set of cognitive and social skills that enable individuals to access, comprehend, evaluate, and apply nutritional information to make healthy food choices and dietary decisions (3). For primary school students, who are in a critical stage for cognitive development and habit formation, a high level of nutrition literacy acts as an intrinsic driver, guiding them to establish scientific dietary patterns, resist unhealthy food marketing, and ultimately achieve lifelong health (4, 5).

Currently, China’s child nutrition landscape presents complex and unprecedented challenges. While economic growth and material abundance have largely eradicated widespread nutritional deficiencies, new problems have emerged simultaneously. The westernization of dietary patterns, increasingly sedentary behaviors, and problematic feeding practices associated with intensive parenting have collectively contributed to the dual burden of “hidden hunger” and overweight/obesity (6, 7). According to research by Dang et al. (8), the prevalence of overweight and obesity among Chinese children and adolescents aged 7–18 years has increased dramatically from 5.3% in 1995 to 24.2% in 2019, implying the severe consequences of poor dietary behaviors during childhood. Traditional nutrition education models, predominantly focused on knowledge dissemination, have demonstrated limited effectiveness in addressing these challenges. Consequently, developing systematic approaches to enhance children’s literacy in translating nutritional knowledge into practical behaviors has become an urgent priority in tackling the current predicament.

Previous studies have established that nutrition literacy in children and adolescents is shaped by a complex interplay of factors. Socioeconomic determinants—particularly parental education, household income, and urban residence—consistently demonstrate positive correlations with nutrition literacy levels (9–11). A paradoxical trend emerges with age: as children gain greater autonomy in food selection and consumption, their nutrition literacy may paradoxically decline, potentially reflecting how increased independence outpaces the development of adequate decision-making competencies (12). Gender differences are also evident, with females generally exhibiting higher nutrition literacy than males (13). Notably, caregiver arrangement represents a significant influence, as children under the care of both parents typically demonstrate better nutrition literacy compared to those raised by single parents or other guardians (14). Furthermore, structured nutritional education interventions have been shown to substantially enhance nutrition literacy (15). These findings collectively illustrated the multifaceted nature of influences on youth nutrition literacy.

Shenzhen City, serving as a pioneering demonstration zone for China’s reform and opening-up policy, provides exemplary research setting for investigating urbanization’s impact on child health, characterized by its rapid economic growth, highly mobile population, and socio-cultural diversity. To systematically address existing gaps in understanding the status and determinants of nutrition literacy among primary school students in the modern city, we applied the Social-Ecological Model (SEM) and concentrating on Nanshan District of Shenzhen City. The SEM provides a framework that health behaviors and outcomes are shaped by a hierarchy of impact factors, ranging from intrapersonal factors (e.g., knowledge, attitudes), to interpersonal processes (e.g., family influences), organizational settings (e.g., school environment), and broader community and societal factors (16, 17). The empirical evidences generated are expected to establish a solid foundation for developing targeted, scientifically sound, and effective nutritional intervention strategies for children in urban settings like Shenzhen.

## 2 Materials and methods

### 2.1 Study participants

In October 2024, this study was executed to target public primary school students in grades 3–5 from Nanshan District, Shenzhen. A multi-stage clustered random sampling method was employed: first, 21 schools were randomly selected from Nanshan District using a random number table; then, within each selected school, one class was randomly chosen from each of grades 3–5 through stratified sampling by grade. A total of 62 classes were included, and all students in these classes were invited to participate in the survey. The study protocol was approved by the Ethics Committee of the Shenzhen Nanshan Center for Chronic Disease Control (No. LL20230034). Informed consent was obtained from their guardians prior to the investigation.

### 2.2 Study methods

A self-administered questionnaire survey was conducted using the “Nutrition Literacy and Dietary Behavior Questionnaire for School-aged Children”, which was developed by the School of Public Health at Peking University (18). The scale has demonstrated good reliability and validity (19, 20).

Nutrition literacy of this questionnaire encompasses four dimensions: (1) Nutrition-related knowledge and concepts (14 items, 31 points); (2) Food selection (12 items for grades 3–4, 24 points, 13 items for grade 5 and above, 26 points); (3) Food preparation (5 items, 10 points); (4) Food intake (15 items for grades 3–4, 30 points, 17 items for grade 5 and above, 34 points). All items used a 5-point Likert scale scored 0, 0.5, 1, 1.5, and 2 points, respectively. Reverse scoring was applied for negatively worded items. Matching questions were awarded 1 point for each correct connection, while multiple-choice questions received 2 points for correct answers. The total scores were 95 points (46 items) for grades 3–4 and 101 points (49 items) for grade 5. To enable comparison across different grades, total scores were converted to a percentage system for analysis. Based on the Nutrition and Health Knowledge Survey (21), a converted total score ≥75 points was defined as “adequate nutrition literacy”.

This study collected information such as gender, grade, primary caregiver, educational level of the primary caregiver, and family socioeconomic status using a basic information questionnaire. Family socioeconomic status was assessed using a questionnaire adapted from the “Family Affluence Scale II (FAS II)” of the “Health Behavior in School-aged Children (HBSC) survey”, which as originally developed in Europe and North America. Through linguistic and cultural adaptation, FAS II has been widely validated and applied in Chinese contexts (22, 23). For this Shenzhen-based study, we made further adaptations to the scale items to account for the distinctive characteristics of this rapidly urbanizing metropolis. Shenzhen maintains a highly developed transportation system, including extensive subways, taxis, and ride-hailing services. Coupled with the wide availability of affordable domestic automobiles nowadays in China, family vehicle ownership has become a weak indicator for differentiating socioeconomic status. Meanwhile, as a pioneer city in mobile internet infrastructure and digital applications, smartphones have largely substituted personal computers for most daily and educational activities, thereby diminishing the value of computer ownership as a valid marker of socioeconomic advantage. Consequently, we retained only two items for the current study: (1) “Do you have your own bedroom?” (Yes = 2 points, No = 0 points); (2) “How many times did your family travel for vacation (away from your usual residence) in the past 12 months?” (None = 0 points, Once = 1 point, Twice = 2 points, More than twice = 3 points). The total score ranges from 0 to 5. Based on the total score, families were categorized into three socioeconomic levels: “Low level” (scores 0–1), “Medium level” (scores 2–3), and “High level” (scores 4–5) (24).

The weight status of children was evaluated in accordance with “Screening for overweight and obesity among school-age children and adolescents” (WS/T 586—2018) (25). Body mass index (BMI) was calculated as weight (kg) divided by height squared (m^2^), based on physical examination data retrieved from the Shenzhen Primary and Secondary School Students’ Health Information Management System.

### 2.3 Quality control

Quality control was implemented following standardized protocols for the survey procedure, questionnaire administration, and workflow. All staff members involved received systematic training prior to data collection. A pre-survey assessment was conducted to evaluate the field investigation capabilities of site investigators. During the survey process, investigators provided uniform instructions regarding response requirements, while simultaneous on-site monitoring and supervision were performed. To enhance data quality and minimize social desirability bias, the students were surveyed in a controlled classroom setting in the absence of teachers, with explicit assurances from investigators that responses were anonymous and non-judgmental. Quality control officers reviewed all questionnaires, verifying and correcting any omissions or errors, and documented the process using standardized quality control forms. Data management was performed using EpiData 3.0 software with a double-data entry protocol, where discrepancies were reconciled through verification with original records. Following data entry, outlier detection and processing were conducted, and questionnaires lacking critical variables were excluded from the final analysis.

### 2.4 Statistical analysis

Following logical checks and data cleaning procedures, statistical analyses were conducted using R-4.5.2 for Windows. Continuous variables were summarized as mean ± standard deviation, while categorical variables were described using frequency counts and percentages. For group comparisons, independent t-tests or one-way ANOVA were applied for continuous variables, and Chi-square tests were utilized for categorical variables. Correlation analyses between nutrition literacy dimensions employed Spearman’s rank correlation coefficient. To identify independent impactors for adequate nutrition literacy (0 = inadequate, 1 = adequate), multivariable logistic regression analysis was implemented, with the independences of gender, grade, BMI, household economic status, primary caregiver, primary caregiver’ education level, participation in nutrition activities and weight monitoring. A two-tailed *p* < 0.05 was considered statistically significant for all analyses.

## 3 Results

### 3.1 Basic characteristics

A total of 2,480 questionnaires were distributed, and 2,423 valid responses were collected and included in this analysis. The sample consisted of 1,254 boys (51.00%) and 1,169 girls (49.00%), with a mean age of 9.82 ± 0.93 years. According to BMI classifications, 22.88% (649/2,423) of the students were overweight or obese. In terms of caregiving arrangements, parents served as the primary caregivers for 84.69% (2,052/2,423) of the participants. Regarding educational background, 77.39% (1,875/2,423) of the primary caregivers held a bachelor’s degree or higher. The study also found that 55.14% students (1,336/2,423) had not participated in any nutrition-related activities (including nutrition education lectures, nutrition knowledge competitions, or school-based nutrition campaigns), while a high proportion (70.37%, 1,705/2,423) reported undergoing regular weight monitoring (see Table 1).

**Table 1:** Sociodemographic characteristics of primary school students in Nanshan District, Shenzhen.

Variable	*N*	Mean/proportion (%)	SD/cumulative frequency (%)
Age	2,423	9.82	0.93
Gender			
Boys	1,254	51.00	51.00
Girls	1,169	49.00	100.00
Grade			
Grade 3	824	34.01	34.01
Grade 4	831	34.30	68.31
Grade 5	768	31.69	100.00
BMI			
Normal	1,774	73.22	73.22
Overweight/obesity	649	26.78	100.00
Household economic status			
High	1,236	51.01	51.01
Medium	321	13.25	64.26
Low	866	35.74	100.00
Primar caregiver			
Parents	2,052	84.69	84.69
Grandparents	328	13.54	98.23
Others	42	1.77	100
Primary caregiver’s education level			
Primary school or below	118	4.87	4.87
Junior high school	153	6.31	11.18
Senior high school	277	11.43	33.61
Bachelor’s degree or above	1,875	77.39	100.00
Participation in nutrition activities			
Yes	1,087	44.86	44.86
No	1,336	55.14	100.00
Weight monitoring			
Yes	1,705	70.37	70.37
No	718	29.63	100.00

### 3.2 Nutrition literacy scores of primary school students

The surveyed primary school students demonstrated an average total nutrition literacy score of 69.93 ± 8.75 points. Analysis across the four dimensions revealed the following mean scores: nutrition-related knowledge and concepts was 22.54 ± 3.31, food selection was 17.66 ± 3.40, food preparation was 6.98 ± 21.56, and food intake was 22.75 ± 3.75. Total nutrition literacy scores varied significantly across several demographic and behavioral factors, notably grade level, household economic status, primary caregiver’s education level, participation in nutrition-related activities, and regular weight monitoring (*p* < 0.05) (see Table 2).

**Table 2:** Nutritional literacy and sub-dimension of nutritional literacy among different sociodemographic characteristics of students, and univariable analysis.

Variable	Option	*N*	Nutrition-related knowledge and concepts	Food selection	Food preparation	Food consumption	Total score	*F/t*	*p*
Gender	Boys	1,254	22.51	17.69	6.97	22.54	69.71	1.287	0.198
	Girls	1,169	22.57	17.63	7.00	22.97	70.17		
Grade	Grade 3	824	22.90	17.85	7.44	22.81	71.00	14.310	<0.001
	Grade 4	831	22.87	17.78	7.01	22.38	70.05		
	Grade 5	768	21.80	17.31	6.47	23.08	68.67		
BMI	Normal	1774	22.53	17.66	6.93	22.73	69.86	0.173	0.841
	Overweight/obesity	649	22.77	17.76	7.14	22.41	70.09		
Household economic status	High	1,236	22.77	17.93	7.06	23.02	70.78	11.989	<0.001
	Medium	321	22.37	17.18	6.95	22.39	68.90		
	Low	866	22.27	17.44	6.89	22.50	69.11		
Primary caregiver	Parents	2,055	22.57	17.69	7.99	22.83	70.06	1.567	0.209
	Grandparents	328	22.35	17.51	6.89	22.38	69.14		
	others	42	23.51	17.82	7.16	21.48	69.97		
Primary caregiver’s education	Primary school or below	118	21.93	17.13	6.71	22.08	67.86	4.560	0.003
	Junior high school	153	22.02	17.23	6.79	22.63	68.67		
	Senior High school	277	22.22	17.71	6.96	22.55	69.35		
	Bachelor’s degree or above	1875	22.67	17.72	7.04	22.83	70.26		
Participation in nutrition activities	Yes	1,087	22.90	17.34	6.93	22.55	69.07	−5.437	<0.001
	No	1,336	22.24	18.05	7.05	22.99	71.00		
Weight monitoring	Yes	1,705	22.75	18.05	7.09	23.26	71.15	−10.790	<0.001
	No	718	22.03	16.73	6.74	21.54	67.05		

*F* for ANOVA (>2 groups); *t* for *t*-test (2 groups).

### 3.3 Correlation analysis among dimensions of nutrition literacy

Spearman correlation analysis revealed statistically significant positive correlations among all four dimensions of nutrition literacy (*p* < 0.001). Specifically, nutrition-related knowledge and concepts demonstrated positive correlations with food selection (*r* = 0.363), food preparation (*r* = 0.225), and food Consumption (*r* = 0.295). Food selection was positively correlated with both food preparation (*r* = 0.198) and food Consumption (*r* = 0.255). Additionally, a positive correlation was observed between food preparation and food Consumption (*r* = 0.261). These findings indicate consistent interrelationships among the different dimensions of nutrition literacy, suggesting the interrelated nature of knowledge, skills, and behaviors in forming a comprehensive system of nutritional competencies.

### 3.4 Univariable analysis of adequate nutrition literacy

The rate of adequate nutrition literacy among primary school students was 30.38% (736/2,423). Univariable analysis revealed several significant associations: students with regular weight monitoring showed a significantly higher rate of adequate nutrition literacy compared to those with irregular monitoring (*χ*^2^ = 61.549, *p* < 0.001). Additionally, third-grade students had the highest rate among all grades (*χ*^2^ = 9.251, *p* = 0.010). Furthermore, students whose primary caregivers were their parents also showed significantly higher rate compared to those cared for by grandparents or others (*χ*^2^ = 7.176, *p* = 0.028) (see Table 3).

**Table 3:** Univariable analysis on adequate nutrition literacy among primary school students.

Variable	Items	*N*	Rate of adequate (%)	*χ* ^2^	*p*
Gender	Boys	1,254	29.74	0.484	0.507
	Girls	1,169	31.05		
Grade	Grade 3	824	33.01	9.251	0.010
	Grade 4	831	31.53		
	Grade 5	768	26.30		
BMI	Normal	1,774	29.48	2.398	0.301
	Overweight/obesity	649	32.82		
Household economic status	High	1,237	34.36	2.504	0.114
	Medium	320	25.63		
	Low	866	26.44		
Primary caregiver	Parents	2,052	31.34	7.176	0.028
	Grandparents	326	24.23		
	Others	45	31.11		
Primary caregiver’s education	Primary school or below	118	22.88	7.360	0.061
	Junior high school	153	30.07		
	Senior high school	277	25.63		
	Bachelor’s degree or above	1,875	31.57		
Participation in nutrition activities	Yes	1,087	25.48	17.292	<0.001
	No	1,336	26.87		
Weight monitoring	Yes	1,705	35.13	61.549	<0.001
	No	718	19.08		

*Adequate = nutrition literacy score ≥75.

### 3.5 Multivariable logistic regression analysis on adequate nutrition literacy

The multivariable logistic regression model revealed that students had significantly higher odds of attaining adequate nutrition literacy if they came from a high-income household (OR = 1.389, 95% CI: 1.139–1.649), participated in nutrition-related activities (OR = 1.346, 95% CI: 1.125–1.611), or regularly monitored their weight (OR = 2.213, 95% CI: 1.784–2.746). Students in Grade 5 had lower odds of nutrition literacy attainment compared to Grade 3 (OR = 0.626, 95% CI: 0.500–0.783) (see Table 4).

**Table 4:** Multivariable logistic regression analysis on adequate nutrition literacy among primary school students.

Variables	Variable assignment	B	SE	*χ* ^2^	*p*	OR (95% CI)
Constant		−1.462	0.419	12.151	<0.001	0.232
Gender	Girls = 0, boys = 1	0.076	0.092	0.681	0.409	1.079 (0.901, 1.292)
Grade	Reference was grade 3					
Grade 4		−0.128	0.108	1.395	0.237	0.880 (0.711, 1.088)
Grade 5		−0.469	0.115	16.718	< 0.001	0.626 (0.500, 0.783)
BMI	Reference was normal					
Overweight/obesity		−0.149	0.103	2.095	0.148	0.862 (0.705, 1.054)
Household economic status	Reference was low					
Medium		−0.045	0.153	0.085	0.771	0.956 (0.709, 1.291)
High		0.328	0.101	10.500	0.001	1.389 (1.139, 1.694)
Primary caregiver	Reference was others					
Grandparents		−0.662	0.365	3.286	0.070	0.516 (0.252, 1.055)
Parents		−0.291	0.344	0.714	0.398	0.748 (0.381, 1.468)
Primary caregiver’s education	Reference was primary					
Junior high school		0.504	0.289	3.044	0.081	1.655 (0.940, 2.916)
Senior high school		0.101	0.265	0.146	0.702	1.106 (0.659, 1.858)
Bachelor’s degree or above		0.365	0.230	2.515	0.113	1.440 (0.918, 2.261)
Participation in nutrition activities	No = 0, Yes = 1	0.297	0.092	10.532	0.001	1.346 (1.125, 1.611)
Regular weight monitoring	No = 0, Yes = 1	0.794	0.110	52.088	<0.001	2.213 (1.784, 2.746)

## 4 Discussion

This large-scale cross-sectional study revealed a moderate level of nutrition literacy among primary school students in grades 3–5 from Nanshan District, Shenzhen City. And several key factors were associated with the adequate nutrition literacy, such as household economic status, participation in nutrition-related activities, and regular weight monitoring. These results underscore the need for comprehensive, multi-level intervention strategies that address the complex interplay of individual, familial, and institutional factors affecting children’s nutritional competencies.

Currently, research on nutrition literacy among Chinese school-aged children remains in a developmental phase, characterized by methodological limitations including inconsistent evaluation tools and lack of standardized cutoff values for determining adequate nutrition literacy. Using the same questionnaire as the present study, a median score of 71.1 was reported among primary school students in Beijing (41.9% for adequate nutrition literacy rate) (12), and 65.4 for school-aged children in Grade 3 to 9 (with 67.0 for Grade 3 to 4, and 66.5 for Grade 5 to 6) across 27 provinces of China (26). While the median of nutrition literacy was 61.68 for middle school students in Chongqing, measured by “Nutrition literacy scale for middle school students in Chongqing” (27). In this study, the level of nutrition literacy (score of 69.93, rate of 30.38%) in primary school students was similar to these findings, suggesting a relatively moderate level of nutrition literacy for urban Chinese primary students.

The observed variations in nutrition literacy can be effectively interpreted through the SEM theoretical framework for this study. Our findings align with this framework, and the following discussion is structured to examine these influences, beginning with the paradoxical role of student grade level (intrapersonal level), and then expanding to encompass the micro-system of the family (interpersonal processes) and the organizational influence of the school.

At the intrapersonal level, the results revealed that third- and fourth-grade students demonstrated significantly higher rates of adequate nutrition literacy compared with fifth-grade students, which was the same as that showed in study of Wang et al. (26). This pattern contrasts with established cognitive developmental theories, as older students exhibit more advanced logical reasoning and information integration capabilities, enabling deeper comprehension and application of complex nutritional concepts such as dietary balance and food composition (28). For lower rate of adequate nutrition literacy in higher grade, a possible explanation is associated with the unique developmental challenges of early adolescence, a critical period for health socialization where cognitive and social priorities shift (29). Additionally, older students often experience greater autonomy and exposure to diverse food environments outside school that does not conform with nutritional guidelines, which might limit the influence of school-based nutrition education received at younger grades (30, 31). Future longitudinal research is needed to disentangle the specific effects of developmental factors, and environmental influences on nutrition literacy during this transition.

Within the micro-system of the family, household economic status emerged as a significant predictor, aligning with global evidence on socioeconomic disparities in health literacy. Higher family economic status may facilitate access to diverse foods and nutrition-related resources. In contrast, the type of primary caregiver (e.g., parents vs. grandparents) was not a significant predictor in our analysis—a finding inconsistent with the earlier study by Young et al. (32). This discrepancy may stem from different types of primary caregiver distribution, i.e., there are less primary caregiver of grandparents in Shenzhen than other cities in China. However, it is important to note that the non-significant variations do not necessarily imply that family caregivers are unimportant. Within the context of China’s prevalent intergenerational parenting model in most cities, grandparents’ feeding beliefs were largely shaped during a period of relative material scarcity, which has led them to express care primarily by ensuring children are “well-fed” with energy-dense, high-fat foods. Consequently, their awareness and practice of modern nutritional principles—such as dietary diversity, salt reduction, and sugar control—are generally limited (33, 34). Moreover, grandparents often exhibit more permissive parenting behaviors, including greater tolerance toward children’s unhealthy snack choices and eating habits (35). These findings highlight the critical importance of incorporating families, particularly primary caregivers, into nutrition intervention strategies. Implementing intergenerational nutrition education programs, such as structured “grandparent classrooms,” would help modernize grandparents’ nutritional knowledge and bridge the generational gap in dietary concepts.

At the organizational level, participation in school-based nutrition activities was positively associated with the attainment of adequate nutrition literacy, a finding consistent with the earlier research by Jakobovich et al. (36). A comprehensive systematic review of school-based interventions targeting FL and NL in primary-school-age children identified that experiential strategies including hands-on food education, school gardening, kitchen classrooms, family cooking and shared activities, and culturally appropriate food practices effectively improved functional skills as well as partial interactive and critical competencies (37). This suggests that students who participate in such activities may have greater opportunities to develop practical nutrition skills and apply nutritional knowledge in real-world contexts. However, the rate of adequate nutrition literacy (30.38%) among the students in Nanshan District was below one third and more than half of the students (55.14%) reported no participation in any nutrition-related activities at school, indicating potential shortcomings in either the coverage, design or implementation of current school-based nutrition education programs. Several factors might explain this gap, such as limited school resources, inadequate teacher training in nutrition education, restrict time allocation for health promotion activities. Additionally, many programs appear to prioritize procedural compliance over substantive engagement, lacking relevance to students’ daily dietary experiences and failing to cultivate interest. The predominance of didactic instruction methods may further contribute to student disengagement and resistance (38). Such limitations not only compromise educational effectiveness but may inadvertently produce adverse outcomes. Also, in this study, regular weight monitoring was positively associated with nutrition literacy in our cross-sectional analysis. Recently, weight monitoring often remains a spontaneous behavior among caregivers and has not been effectively integrated with systematic school nutrition education. Most caregivers’ approaches to weight monitoring remain limited, failing to guide children in understanding the crucial balance between weight management, overall dietary patterns, and physical activity. Consequently, children may develop a superficial focus on body weight metrics alone, potentially leading to weight-related anxiety and psychological burden (39). Healthy weight management should derive from active lifestyle adoption rather than passive monitoring. This should involve widespread adoption of experiential and inquiry-based learning modalities, including physical activities, school gardening programs, culinary workshops, and interactive food label analysis, enabling students to discover the practical value of nutritional knowledge through firsthand experience (37, 40, 41) and integrating weight monitoring into comprehensive health education. This holistic approach will help students establish the correct understanding that “healthy weight results from balanced nutrition and appropriate physical activity”.

This study has several limitations. First, the cross-sectional design precludes definitive causal inferences regarding the identified relationships. Second, the sampling frame was restricted to a single geographic district, which may limit the generalization of findings to other regions with different socioeconomic and cultural contexts. Third, the assessment of nutrition literacy relied primarily on self-reported questionnaire data, which may be subject to social desirability and recall biases. Children may provide their answers according to the teachers or parental attitudes. Despite our on-site protocols designed to ensure independent and private responding, these biases could lead to an overestimation of nutrition literacy, particularly in behavioral domains, and may affect the strength of the observed associations. Future research would benefit from incorporating multi-informant designs or objective measures. Despite these limitations, this investigation provides valuable insights into the current status of nutrition literacy and its key determinants among primary school students in Shenzhen. The findings establish an important evidence base for developing targeted interventions and offer methodological considerations for future research in this emerging field. Future longitudinal studies incorporating objective assessment methods and more diverse sampling approaches would help validate and extend these findings.

## 5 Conclusion

This study demonstrates that nutrition literacy among primary school students in Nashan District, Shenzhen City requires substantial improvement, with rates of adequate nutrition literacy significantly influenced by grade level, household economic status, participation in nutrition-related activities, and regular weight monitoring. Future interventions should adopt an evidence-based, systematic approach that moves beyond traditional knowledge-transmission models. A comprehensive strategy should include: (1) developing student-centered learning experiences that connect nutrition knowledge to real-life contexts; (2) establishing robust home-school collaboration mechanisms to engage caregivers; and (3) implementing experiential activities that bridge the gap between knowledge and practice. Particular attention should be given to optimizing school-based health programs to ensure they produce meaningful outcomes. This multifaceted approach will better support the transformation of nutritional knowledge into sustainable healthy behaviors, ultimately enhancing the effectiveness of school health education initiatives.

## Data Availability

The raw data supporting the conclusions of this article will be made available by the authors, without undue reservation.
